# Cryopreservation of Agronomic Plant Germplasm Using Vitrification-Based Methods: An Overview of Selected Case Studies

**DOI:** 10.3390/ijms22116157

**Published:** 2021-06-07

**Authors:** Cesar Augusto Roque-Borda, Dariusz Kulus, Angela Vacaro de Souza, Behzad Kaviani, Eduardo Festozo Vicente

**Affiliations:** 1 School of Agricultural and Veterinarian Sciences, São Paulo State University (UNESP), Jaboticabal 14884-900, SP, Brazil; cesar.roque@unesp.br; 2 Laboratory of Ornamental Plants and Vegetable Crops, Faculty of Agriculture and Biotechnology, UTP University of Science and Technology in Bydgoszcz, Bernardyńska 6, 85-029 Bydgoszcz, Poland; 3 School of Sciences and Engineering, São Paulo State University (UNESP), Tupã 17602-496, SP, Brazil; angela.souza@unesp.br (A.V.d.S.); eduardo.vicente@unesp.br (E.F.V.); 4 Department of Horticultural Science, Rasht Branch, Islamic Azad University, Rasht 4147654919, Iran; b.kaviani@yahoo.com

**Keywords:** cryoprotectant, droplet-vitrification, encapsulation-dehydration, encapsulation-vitrification, gene banks, genetic integrity, molecular markers, somaclonal variation, vitrification

## Abstract

Numerous environmental and endogenous factors affect the level of genetic diversity in natural populations. Genetic variability is the cornerstone of evolution and adaptation of species. However, currently, more and more plant species and local varieties (landraces) are on the brink of extinction due to anthropopression and climate change. Their preservation is imperative for the sake of future breeding programs. Gene banks have been created worldwide to conserve different plant species of cultural and economic importance. Many of them apply cryopreservation, a conservation method in which ultra-low temperatures (−135 °C to −196 °C) are used for long-term storage of tissue samples, with little risk of variation occurrence. Cells can be successfully cryopreserved in liquid nitrogen (LN) when the adverse effect of ice crystal formation and growth is mitigated by the removal of water and the formation of the so-called biological glass (vitrification). This state can be achieved in several ways. The involvement of key cold-regulated genes and proteins in the acquisition of cold tolerance in plant tissues may additionally improve the survival of LN-stored explants. The present review explains the importance of cryostorage in agronomy and presents an overview of the recent works accomplished with this strategy. The most widely used cryopreservation techniques, classic and modern cryoprotective agents, and some protocols applied in crops are considered to understand which parameters provide the establishment of high quality and broadly applicable cryopreservation. Attention is also focused on the issues of genetic integrity and functional genomics in plant cryobiology.

## 1. Introduction

The phylogenetic resource is defined as a genetic material of plant origin that has real (economic) or potential (scientific and cultural) value for food and agriculture [1]. The genetic diversity of plants has been reduced in the past few decades and replaced by the uniformity of crops in the so-called genetic erosion process. This crop uniformization makes agrotechniques easier but, consequently, less profitable species and varieties are abandoned. The loss of genetic variability limits the plants’ capability to respond to environmental changes and favors the appearance of new pests or diseases. It also makes further breeding programs more challenging. The importance of biological diversity conservation was recognized internationally in 196 countries and has generated a treaty that also includes the sustainable use of its components and fair and equitable participation in the benefits derived from the use of plant resources [2].

According to Lambers [3], one of the biggest concerns of biologists is that the rapid warming rates projected for the planet could lead many species to perish. Climate change is altering environmental niches, forcing species to shift their habitat range [4]. They can suffer from extinction if the specific habitat disappears or becomes inaccessible, due to geographical barriers or the species’ inability to disperse. Previous studies have provided regional or taxonomic estimates of biodiversity loss with a climate change contribution of 54%, making it difficult to neglect the severity of this problem [3,5].

There is great interest in understanding how species can respond to climate change, but forecasts show divergences and incipient data. As of 2015, more than 130 studies have been carried out to identify the level of risk that climate change represents for species and the specific traits and characteristics that contribute to the risk. If climate change continues at the current rate, one in six species could face extinction [6]. Several regions, including South America and Oceania, face an increased hazard due to temperature change, generating global warming, which consequently causes the melting of the glacier poles, destabilizing various ecosystems, and extinguishing natural resources [7]. The predictions for the 1980s were that about 20,000 species of higher plants, medicinal plants, and forest trees would be in danger of extinction [8]. However, over the years, this problem has been exacerbated due to indiscriminate deforestation, overcollection, intensive farming, and pollution, causing a continuous depletion of genetic variability.

Due to all these factors, germplasm banks were created that store different plant materials, to maintain a collection of the possible genetic variations existing in the world [9]. This review aims to analyze the current strategies of biodiversity protection with particular attention focused on cryopreservation as a method of long-term storage of plant material and some of its applications made in agronomy in recent years. Attention is also focused on the issues of genetic integrity and genomics in plant cryobiology.

## 2. Preservation of Plant Genetic Resources

**Figure 1:**
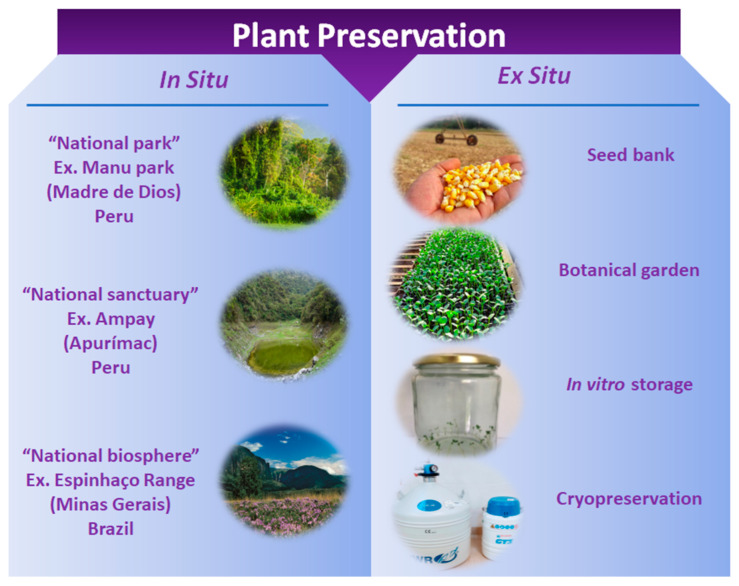
Basic strategies of plant preservation.

Genetic resources of plants can be conserved in their natural habitat (in situ) or other sites (ex situ), depending on the storage capacities or resources available and the characteristics of the species (Figure 1). In situ is the process of protecting a species, animal, or plant threatened by extinction in its natural habitat (in place/on-site), acting or not in the habitat itself, or defending this species from its predators. In situ protection is mainly provided by nature reserves and national parks. The benefit of preservation in situ is that recovering populations are maintained in the environment in which their distinct properties develop. Preservation ex situ (off-site) can be used in part or in the entire population when preservation in situ presents insurmountable difficulties, usually resulting from a lack of complete control over the many factors which influence the survival of individuals and, therefore, the genetic makeup of the conserved population [10,11]. Different ex situ systems emerge as a complementary measure to preservation in situ, mainly aimed at protecting genetic material and ensuring its maintenance over time, in viable conditions and with original genetic characteristics [8,12].

The germplasm of species with orthodox seeds (i.e., dehydration-tolerant) can be conserved in various ways, such as field collections, greenhouses, seed banks, or in vitro. The in vitro preservation methods are carried out by vegetative propagation and slow-growth storage [13,14,15,16] and cryopreservation [17]. As for species with recalcitrant seeds (with low viability), it is necessary to resort to cryopreservation or maintain the vegetative material in field collections or in vitro culture [18].

***Field collections:*** it is a simple, well-known method of biological diversity preservation. It can be carried out in situ in areas where it occurs naturally, trying to maintain the diversity of living organisms, their habitats, and the interrelationships between organisms and the environment [19,20]. Alternatively, field collections may also refer to ex situ conservation if they are set off-site the natural habitat of a species, e.g., in plots, parks, gardens, etc.

In vegetatively propagated crops, such as potato (*Solanum tuberosum* L.), cassava (*Manihot esculenta* Crantz), and yam (*Dioscorea*), to maintain genetic stocks (tubers and roots), they need to be stored and grown annually in nurseries. This is time-consuming and problematic, as well as requiring a lot of space and work. Moreover, the material can be exposed to pests and pathogens and there is often a risk of germplasm loss [21]. Therefore, researchers are searching for complimentary preservation methods.

***Seed banks:*** are used for the storage of high-quality orthodox seeds (or pollen) in plastic bags, vials, or glass jars in a controlled environment (low temperature and humidity). Working and active collections are refrigerated (at 5 °C). Longer storage of base collections is possible after (deep)freezing of seeds (−20; −80 °C). Depending on the container used, storage temperature, and seed viability, the collections must be periodically regenerated, and the produced plants are further selected to replenish seed stocks. Consequently, most gene banks also maintain field collections [17].

***In vitro preservation:*** in vitro storage is an essential part of the overall strategy for the preservation and exchange of plant genetic resources worldwide [22]. In vitro germplasm banks conserve plant genetic resources under controlled aseptic conditions and involve various cell and tissue culture techniques, as well as synthetic seeds [23]. In vitro culture of slow growth is widely used to obtain medium-term storage of tropical species explants, crops with vegetative propagation (e.g., potato, banana, cassava), or endangered species [24]. This method is also known as ‘reduced or minimal growth’ because it is based on decreased cell division and plant metabolism. Its objective is to increase the longevity of in vitro cultures without genetic alterations. Therefore, there is no total stoppage of cellular processes, but a decrease in the speed with which they occur and therefore in the frequency of subculturing plants to the fresh culture medium to one per several months or even years, depending on the species [18]. This growth reduction is usually obtained by decreasing the temperature and light intensity in the growth room and/or modifying the composition of the culture medium by increasing the concentration of osmotic agents or growth retardants.

## 3. Cryopreservation

Cryopreservation is an important tool for storing germplasm, developed in recent decades and already implemented in current germplasm banks, e.g., CIAT, Colombia; EMBRAPA, Brazil; CRI, Czech Republic; InHort, Poland; NARO, NIAS, NCSS, Japan; and many others [9]. This method consists of the use of ultra-low temperatures (−135 °C to −196 °C) of liquid nitrogen (LN), which allows cells to maintain their viability and genetic stability. Cellular aging is interrupted due to nearly complete paralysis of its metabolism, physiological and biochemical processes [25,26].

The first report of successful cryopreservation of plant cells grown in vitro was from Quatrano [27] from hypocotyl-derived callus of aseptically germinated flaxseeds (*Linum usitatissimum* L.), using dimethyl sulfoxide (DMSO) as a protector against disruptions caused by cooling. Until now, it was known that cells with low water content, such as pollen, seeds, and dormant tissues of stress-tolerant species, may be subjected to the temperature of LN without suffering lethal damage. However, plant cells with higher water content present considerable problems due to the risk of ice crystal growth causing cell bursting. Sakai [28] demonstrated, for the first time, that successful cryopreservation involves the use of cryoprotectants and controlled cooling rates, and that this method could be adapted for use with cells from a wide variety of plant species [29].

Among the several reasons that lead to the application of cryopreservation, one can mention (1) facilitation of hybridization between plants that flower at different times; (2) hybridization between plants that grow in different and distant places; (3) reduction in the spread of diseases by pollination vectors, and (4) long-term maintenance of high-quality germplasm [30]. Cryopreservation minimizes the risk of variation occurrence, both somaclonal variation (non-sexual) and gametoclonal variation, which may occur in tissue culture or field collections, respectively. Therefore, it is possible to obtain cultures with vegetative propagation and genetic uniformity of the clones.

In the particular case of recalcitrant seeds, which are sensitive to low temperature and humidity, the embryo is short-lived [31]. Several species of recalcitrant seeds and agricultural importance are known, such as coconut tree (*Cocos nucifera* L.), palm oil (*Elaeis guineensis* Jacq.), coffee (*Coffea arabica* L.), mango (*Magnifera* L.), chestnut (*Castanea sativa* Mill.), and walnut (*Juglans* L.) [32,33,34], in which germplasm cannot be maintained through seedbanks. The cryopreservation of whole embryos, their segments, or vegetative parts of plants (e.g., shoot tips) can help to overcome this eventuality [35,36].

According to Engelmann and Takagi [37], classic cryopreservation, or controlled/slow/two-step cooling, and newer techniques based on rapid cooling are recognized as the two possible methods for long-term storage (Table 1). The first is based on slow cooling of explants (0.1–2.0 °C·min^−1^) down to −40 °C (or less often −20 °C); then, fast cooling by immersion in LN. There is a wide variety of LN freezers, automatic and manual, in which the speed of cooling and rewarming can be regulated. The disadvantages of this method include the complexity of the operation, the use of expensive and sophisticated equipment that controls the cooling rate, and the formation of ice crystals in extracellular solutions, causing water loss by cells in the so-called freeze-dehydration process [38,39]. The second cryopreservation method is based on vitrification procedures, in which the solidification or vitrification of plant tissue solutions during the ultra-fast cooling process occurs. Vitrification is a physical process in which an aqueous solution solidifies into a metastable amorphous solid known as the glass. In vitrification, the solidification of plant tissue solutions (both intracellular and extracellular) occurs by an extreme increase in their viscosity during the cryoprotective and ultra-fast cooling processes [21]. In this state, the formation of ice crystals is inhibited or minimized [40,41]. The extreme viscosity of the glass interrupts all chemical reactions that require molecular diffusion, thus allowing the stability and latency of explants over time [42]. To obtain this state, the water content reduction and osmoprotection of the explants with sucrose and/or glycerol are often required, although some plant tissues can be cryopreserved just by physical partial desiccation, without the use of sucrose and/or glycerol (e.g., seeds, pollen, embryonic axes) [43]. After lowering the water content of the intra and extracellular solutions, rapid cooling in LN is performed.

## 4. Plant Material Used for Cryostorage

The physiological state of the mother plant is a key factor for the success of cryopreservation [44,45]. The cryopreservation efficiency depends on the position, age, and size of the explant. In vitro-derived shoot tips or apices are the most common explant types used for this purpose. Both small (1 to 2 mm in length) and intermediate shoot tips or axillary buds (3 to 4 mm) can achieve percentages of recovery comparable to the explants of non-cryopreserved controls. This phenomenon, however, is not observed in larger meristematic explants (5 to 6 mm), which show a strong decrease in the percentage of recovery [46]. Occasionally, other explant types are also used for LN storage. Wang et al. [47] reported 56%, 72%, and 32% shoot regrowth in cryopreserved shoot tips taken from in vitro shoots, adventitious buds regenerated from stem discs, and field-grown bulbs in onion (*Allium cepa* L.), respectively. Chen and Wang [48] developed a cryopreservation protocol for cell suspensions and protoplasts of carrot (*Daucus carota* L.), while Fábián et al. [49] used wheat (*Triticum aestivum* L.) egg cells excised from the ovaries. Carmona-Martín et al. [50], on the other hand, achieved 42–84% recovery of asparagus (*Asparagus officinalis* L.) when rhizome buds were used. Root explants isolated from in vitro-grown plants, as well as adventitious and hairy root cultures, have been recently used in several cryopreservation protocols, including alfalfa (*Medicago truncatula* Gaertn.) [51] and vanilla (*Vanilla planifolia* Andrews) [52]. They attract both scientific and commercial interest as an alternative source of plant-derived bioactive compounds with potential application in pharmaceutical, cosmetic, and natural health product industries [53].

## 5. Cryoprotective Agents (CPAs)

During cryopreservation, many events occur that modify the molecular behavior of water and solutes. Thus, it is a challenge to avoid cytotoxicity and keep plant cell metabolism stable during and after LN storage [54]. When cooling dilute aqueous solutions, thermo-dynamic and physical events occur in different temperature ranges, leading to nucleation. The heterogeneous nucleation occurs in biological systems when ice crystals are formed (about −5 to −20 °C); the homogeneous nucleation process (at less than −40 °C) takes place when this set of molecules self-associate and form nucleated ice [55]. The presence of ice crystals is a threat to cell membranes integrity, as the volume of ice is greater than that of water at the liquid phase. Therefore, nucleation and crystallization usually must be avoided to make the cells to be processed for storage at deep cryogenic temperatures and to be recovered with high levels of appropriate functionality [55].

Vitrification is the process that modifies the viscosity of a solution to avoid nucleation and the formation of ice crystals at low temperatures [54]. To achieve this goal, it is necessary to use various substances, including CPAs, that can reduce the impact of cooling on plant cells [56]. Cryoprotective agents are used to increase osmolarity in the plant tissue and reduce the content of unbound water. Thus, CPAs are capable of modifying phase changes during cooling. CPA solutes possess a wide range of metabolic and biophysical effects. All these thermo-dependent events of molecular interaction, diffusion, and heat flow can be studied using Fourier-transform infrared spectroscopy (ATR-FTIR), scanning electron cryomicroscopy (CRYO-SEM), differential scanning calorimetry (DSC), and thermogravimetric analysis (TGA); however, there are still many of these aspects that are not fully understood [55].

Depending on their properties and functioning, two groups of CPAs can be distinguished, penetrating and non-penetrating.

### 5.1. Penetrating CPAs

Penetrating CPAs are low molecular weight compounds that are permeable through the cell membrane. They act essentially in the protoplast by replacing intracellular water before and during cooling and decrease the freezing point of water, which, combined with a slow or rapid cooling rate, reduces the formation and growth of ice crystals [55]. Penetrating CPAs also function by forming hydrogen bonds with biological molecules as water molecules are displaced. This is important for proper protein and DNA function [57,58]. The molecular weights of CPAs vary between 32 and 212 Da and the most used penetrating CPAs are glycerol, DMSO, and 1,2-propanediol (propylene glycol or PG). 

### 5.2. Non-Penetrating CPAs

Non-penetrating CPAs are substances of high molecular weight that form hydrogen bonds with water. Due to the large size of molecules, they cannot penetrate the cell or enter it with extreme difficulty, reducing ionic forces outside the cell and moderately decreasing the formation of ice crystals. Non-penetrating CPAs include carbohydrates such as polysaccharides, sucrose, and glucose being the most used, or sugar alcohols (mannitol, sorbitol), which are effective due to their osmotic power and thus promote rapid cellular dehydration. To fully express their characteristics, non-penetrating CPAs are usually mixed with penetrating cryoprotectants [59,60].

The addition of the cryoprotectant generates osmotic stress in the cells, as it increases the osmolarity of the medium. Cells are initially dehydrated to compensate for the osmotic force induced by the presence of CPA and then rehydrated after LN storage and rewarming of samples. The definition of the biophysical parameters of each cell type and the study of the interaction with CPA during the cooling and rewarming of the explant must be defined to establish the physical limits that guarantee the survival of the cells [61]. Differential scanning calorimetry (DSC) analyses can be used to help reveal the presence of intracellular ice within explants at key steps during the cryopreservation procedure [62].

## 6. Osmoprotective Solutions

Osmoprotective solutions are preparations with a mixture of low or moderately concentrated penetrating and non-penetrating CPAs. The most common osmoprotective solution used in plant cryobiology is the so-called loading solution 1 (LS). It was developed by Nishizawa et al. [63] and is composed of 2.0 M glycerol and 0.4 M sucrose. Other loading solutions are also known (composed of sucrose, glycerol, DMSO, and/or ethylene glycol (EG) in various concentrations and combinations), although they are less popular (Table 2) [64]. The function of LS is to biophysically prepare the plant specimens to osmotic stress by exposure to a moderately concentrated osmoticum, before severe dehydration with highly concentrated CPAs [65]. Sucrose acts as a protector of the cytoplasmic membrane, or plasmalemma, against dehydration caused by freezing [66]. However, some authors concluded that using only sucrose for dehydration does not guarantee optimal cryopreservation, since in certain plant samples the water transfer mechanism is slower or because complete dehydration could fatally alter the metabolism of plant tissues [66,67,68]. Glycerol neutralizes osmotic stress due to its slower permeable effect on the cell membrane compared to other cryoprotectants, allowing water to escape and partially dehydrate the cell [58,69]. This phenomenon occurs when the conditions of the extracellular environment are hypertonic, that is, the external concentration is greater than that existing inside the cell. For this reason, water inside the vacuole flows into the hypertonic medium (osmosis) and the cell is dehydrated, reducing its size. Under suboptimal conditions, this event can cause the plasma membrane to irreversibly separate from the cell wall. This phenomenon is called permanent plasmolysis and leads to cell necrosis as it cannot return to its normal state. Another type of plasmolysis, the incipient, refers to when the plant cell loses water but can return to its natural state [70]. This type of plasmolysis is essential to obtain when developing a cryopreservation protocol.

Despite the importance of loading treatment and induction of osmotolerance in plant cryopreservation, to ensure high survivability of explants, it is necessary to further treat samples with a plant vitrification solution (PVS), thus leading to vitrification [71].

## 7. Plant Vitrification Solution

PVS solutions are used in all modern cryopreservation techniques due to the osmotic characteristics and protective effects mentioned in the previous sections. PVS at low temperatures increases its viscosity, forming an amorphous glassy state, without ice formation [58]. There are many variations of the PVS solution, of which the best known are PVS1 [74], PVS2 [75], PVS3 [72], and PVS4 [37,76]. The composition of each PVS is shown in Table 3. The solution comes into contact with the cell forming PVS solute compounds modifying the glass transition temperature, and/or with the bound water, which prevents the formation of intercellular ice crystals [28,77].

DMSO is a common solvent in most PVS: bipolar, water-soluble, and aprotic (it has no O―H nor N―H bonds, so it does not donate or receive protons). Its cryoprotective action is mainly attributed to its ability to prevent the excessive accumulation of electrolytes and other substances during the cooling process. Moreover, its low molecular weight allows rapid entry through the cell membrane, in addition to modulating the stability of the phospholipid bilayer. Electrostatic interactions of DMSO with phospholipids have been suggested, appearing to be critical for membrane cryoprotection [81,82].

Ethylene glycol (EG) is widely used as a cryoprotectant, as it has a high coefficient of cell permeability. An important advantage of EG is that it prevents tissue superhydration after the rewarming of samples. Superhydration refers to the unwanted phenomenon of uncontrolled cell expansion after their inoculation on the medium with lower osmoticum concentration, compared to the preculture medium and PVS. This effect is usually observed if glycerol or DMSO are used. One should keep in mind that all treatments with EG must be performed rapidly due to this compound being more toxic than glycerol [83].

Polyethylene glycol (PEG) is a polymer widely used in cryopreservation [84], being a non-penetrating cryoprotectant that, in addition to inhibiting the growth of ice crystals, prevents plant material from suffering injuries caused by cold due to the increase in tonicity of PVS [85]. Evidence indicates that PEG works well in association with DMSO, increasing the percentage of viable explants [21] or with trehalose derivatives, which can adhere to PEG and modify the thermodynamic events by replacing water on the surface of the plant’s cell. It has been shown that short-chain PEGs are particularly effective in producing this effect [85].

## 8. Modern CPAs

Recently, nanosized liposomes from a complex of vegetable (soybean) and animal (egg yolk) phospholipids have been used as a cryoprotectant, being highly efficient in inducing tolerance to the thermal shock produced by storage in LN, especially in synergy with encapsulation in sodium alginate. Autoclaving did not demonstrate any influence on the cryoprotective efficiency of the liposome mixture; however, it should be used fresh as long-term storage resulted in the loss of its beneficial properties [86].

Wheat proteins (such as WCS120, TaTIL, WCS19, and TaIRI-2), obtained from a crude wheat extract were successfully used to improve cryopreservation efficiency and could provide a promising alternative to DMSO [87]. Wang et al. [88] used fish antifreeze protein AFP-I in cryopreservation of rice (*Oryza sativa* L.) embryogenic suspension cells. The positive or negative effect of AFP-I on the viability of cells during the vitrification method depended on the concentration of AFP and their interaction with other CPAs.

Gold and zinc nanoparticles (NPs) are being studied as promising structures that can help protect animal [89] and human cells [90,91] during cryopreservation due to their small size and high thermal conductivity [92]. Nanoparticles can transport heat flux and cause thermal equilibrium with the environment within 10–200 ps [93]. Therefore, their application could significantly accelerate the thermodynamic events during the cooling and rewarming of plant material and prevent the formation of lethal ice crystals. Some other research report NP addition into the culture medium as an effective growth promoter during micropropagation (see *post*-cryopreservation section). However, few studies are reporting the use of NPs as cryoprotective agents in plants. Montalbán et al. [94] used silver nanoparticles (AgNPs) in the culture medium and the preservation solution for deep-freeze storage (at −80 °C) of embryogenic cell lines of *Pinus radiata* D.Don with a recovery level above 75%. Ren et al. [95] found that single-wall carbon nanotubes (SWCNTs) added to the PVS improve cell survival rate and reduce oxidative injury in cryopreserved embryogenic callus of *Agapanthus praecox* Willd. Kulus and Tymoszuk [96], on the other hand, reported that gold nanoparticles (AuNPs) added into the alginate bead matrix improve the survivability of LN-recovered shoot tips of *Lamprocapnos spectabilis* (L.) Fukuhara by even 20%. Future studies should focus on the utility of other nano-colloids, such as copper and titanium, in cryopreservation studies, particularly when added into the LS and PVS. Currently, at the UTP University of Science and Technology in Bydgoszcz, Poland, a research project is being conducted aiming to evaluate the usefulness and influence of silver and platinum nanoparticles, applied at various concentrations during different steps of a cryopreservation procedure, on the development and widely investigated stability of LN-derived plant material (at the structural, metabolic, (cyto)genetic, phenotypic, and biochemical levels).

## 9. Cryopreservation Methods

**Figure 2:**
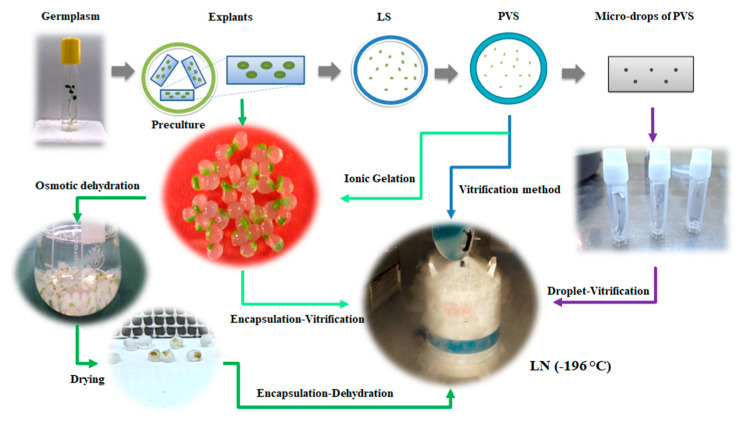
Most common modern cryopreservation methods.

As stated previously, cryopreservation methods can be classified according to the speed of cooling in protocols. Not only does the cooling rate affect the formation rate and size of intracellular and extracellular ice crystals, it also can impact solution effects that occur during the cryopreservation process. Rapid cooling minimizes solute concentration effects (because ice forms uniformly), but it maximizes intracellular ice formation (as water does not have time to migrate out of the cell). Slow cooling creates the opposite result by maximizing water loss from the cell and minimizing intracellular ice formation, but it increases solution effects (www.thermofisher.com, access date 6 June 2021). The most common methods of rapid cooling-based cryopreservation are vitrification, encapsulation-dehydration, encapsulation-vitrification, and droplet-vitrification (Figure 2).

### 9.1. Vitrification

The first step is based on the explant treatment in a preculture medium (PC), which usually contains salts from the MS medium [97], a gelling agent (agar or phytagel), and sucrose at high concentration (usually 6–9% *w*/*v*). Abscisic acid (ABA) and/or proline may also be added. The purpose of this step is to enhance antioxidant metabolism and promote tolerance to stress, especially dehydration [98]. An additional cold-hardening step before or during preculture at 4 °C (or alternation of day/night temperature regimes) for 1–3 weeks may be effective for improving the *post*-thaw survival of some temperate and tropical species, e.g., potato [99,100]. The importance of this step was well demonstrated in kiwi (*Actinidia chinensis* Planch.), where shoot tips that were harvested from cold-hardened plantlets and pre-treated with sucrose and ascorbate showed 40% recovery against 0% in cryopreserved shoot tips that were not pre-treated [101].

The second stage consists of treating the samples with a loading solution (LS), usually for 20 min. This step is followed by direct exposure to PVS, used in the third stage [98,102]. The duration of PVS treatment varies depending on the species, explant type, and PVS itself. PVS containing DMSO require a shorter use (20–40 min) than those without it (even up to 180 min) [58,98]. Finally, the dehydrated explants are placed in 2 mL cryovials and plunged in LN [102].

The first vitrification study was carried out in a particular orange cultivar native to Brazil, commonly known as ‘laranja-umbigo’ (*Citrus sinensis* Osb. var. Brasiliensis Tanaka). In the aforementioned study, the nucellar somatic cells were dehydrated in the PVS2 solution, using a simple methodology with a short execution time (60% PVS2 at 25 °C for 5 min, then chilled PVS2 at 0 °C for 3 min), providing the regeneration level of up to 80% [28,75]. This technique was applied to several other plant species of agronomic interest (Table 4).

### 9.2. Encapsulation-Dehydration

As some plant species do not withstand the harsh conditions of cryopreservation and PVS treatment, a methodology was developed in which the explant follows an encapsulation process by ionic gelation. In this technique, an explant embedded in a drop of sodium alginate (2–3% *v*/*w*), the most studied encapsulating polymer, is placed with a pipet in a cationic solution, such as 100 mM calcium II chloride (CaCl_2_), for approximately 30 min to form protective capsules for the explants. A carrier solution (glycerol and/or sucrose) is also usually added to sodium alginate. These encapsulated explants are dehydrated—osmotically in a series of sucrose solutions (with increasing concentration; up to even 1.0 M), and physically with silica gel or in a laminar flow chamber (to a final level of 10–30% of the initial moisture content)—before immersion in LN [83,103].

### 9.3. Encapsulation-Vitrification

This is a method that combines elements of vitrification and encapsulation-dehydration techniques. The alginate beads are loaded with a sucrose-glycerol solution, dehydrated with PVS, and immersed directly in LN. For rewarming, the capsules are quickly rewarmed in a water bath. The PVS solution is removed from the capsules by washing with sucrose solution and then placed in MS medium for explant recovery [83,104]. In contrast to the encapsulation-dehydration method, dehydration with PVS is faster and easier to control than drying in laminar flow or with silica gel [102].

### 9.4. Droplet-Vitrification

The droplet-vitrification is a modified vitrification technique, which means that the first stages (preculture, LS and PVS treatments) are identical as described in the Vitrification method section. What sets the methods apart is the cooling and rewarming mode of the explant. In the droplet method, cooling is performed in an ultra-fast manner, where the explant trapped in a micro-drop (3–6 µL) of PVS solution is placed on aluminum sheets to be immersed directly in LN. In this process, a thermal drop of up to 1000 °C min^−1^ occurs. On the other hand, during rewarming, foil strips with explants are placed in a sucrose solution and left at room temperature for about 20–30 min, favoring devitrification. Thus, the damage caused by the crystallization of the solution is minimized without compromising the viability of the cells. The main interest of this method is the possibility of achieving very high rates of cooling and/or rewarming due to the low volume of cryoprotectant medium in which the explants are placed and the favorable thermal conductivity of aluminum [44,58].

### 9.5. Cryo-Plate Techniques

The cryo-plates techniques were developed by Yamamoto et al. [105] and Niino et al. [106], who adapted the modified vitrification method to provide stability and greater tolerance of the explant to sudden changes in temperature. The concept of the techniques is based on aluminum microplates containing several oval wells. The explants attached to the cryo-plates with alginate solution are then dehydrated. The V-cryo-plate is based on PVS dehydration, while the D-cryo-plate method is based on air dehydration in the laminar flow chamber for a controlled time. The techniques facilitate the handling of cryopreservation and rewarming procedures and minimize the risk of tiny explants loss and their mechanical injury [107,108,109].

### 9.6. Vacuum-Infiltration-Vitrification

The vacuum-infiltration-vitrification (VIV) technique was developed by Nadarajan and Pritchard [110]. The use of a vacuum during explant incubation in a CPA allows for its increased penetration, and therefore reduces the total duration of the protocol. Another advantage of this approach is the reduction of temperature dependency for cryoprotectant application. VIV cryopreservation also overcomes the problem of specimen heterogeneity (mass, volume, oil composition, and thermal properties). Nonetheless, the technique is rarely used.

### 9.7. Post-Cryopreservation

*Post*-cryopreservation treatments are equally as important as the *pre*-cooling steps to obtain satisfying (at least 40%) explant recovery after cryostorage. Rewarming is performed either at room temperature (as in the case of droplet-vitrification technique) or quickly in a 35–40 °C water bath to avoid ice recrystallization (aggregation of smaller crystals into bigger ones).

PVS are highly effective when performing dehydration or binding to water, preventing it from crystallizing; however, some components of these solutions are toxic to the cell (e.g., DMSO). Consequently, it is necessary to completely remove the PVS solution from the biological material and to secure the cells against uncontrolled rehydration after LN storage [111]. Therefore, after rewarming, the explants are washed with a concentrated sucrose solution, usually liquid MS salts with 1.2 M sucrose for 2 × 1.5 min, the so-called Sakai’s unloading solution (RS) [28]. Maintaining the temperature of the cells at 0 °C during the de- and rehydration processes increased cell survival in wheat [49].

The selection of an appropriate recovery medium plays a critical role in plant regrowth by controlling totipotency, growth, and development of cells and tissues. It is recommended to perform the recovery culture of non-encapsulated explants on a medium with increased osmotic potential and with an addition of appropriate plant growth regulators (PGRs), such as gibberellic acid (GA) during the first few days of recovery [112]. Recent studies demonstrated a higher efficiency of *meta*-topolin (*m*T), a non-conventional cytokinin, than N^6^-benzyladenine (BA) when used during the recovery phase in the LN-derived lateral buds of hazelnut (*Corylus avellana* L.) [113]. Conventional cytokinins, such as BA, are reported to cause certain morpho-physiological, anatomical, and biochemical disorders [114,115]. *Meta*-topolin varies from BA in the conversion of its major metabolic product O-glucoside, which translocates quickly to different parts of the explants [116]. By such means, *m*T alleviates in vitro-influenced disorders such as leaf senescence, hyperhydric shoots, and shoot apex and leaf necrosis [115,117,118].

Physical conditions in the growth room should also be considered. Light quantity and quality (i.e., its intensity, photoperiod, and spectral composition) affect morphogenetic responses of in vitro plants. Modification of light spectra both before LN storage and during recovery after cryopreservation improves survival and recovery. Yoon et al. [45] reported that several potato cultivars grown under high light intensity (140 nmol m^−2^ s^−1^) prior to cryopreservation resulted in significantly higher *post*-cryopreservation recovery. Edesi et al. [119] showed that blue light promoted growth potential, photomorphogenesis, and subsequent survival after cryopreservation of potato clones, while Mølmann et al. [120] showed that the red light produced strong inhibition of sprout elongation even at low irradiances 10–100 nmol m^−2^ s^−1^ in the same species. Nonetheless, the effect of modified light conditions on cryopreservation efficiency is still not well studied.

Successful recovery appears to be dependent upon the presence of antioxidant protection from reactive oxygen species (ROS), often occurring after cryostorage [121]. The addition of vitamin additives into the culture medium such as ascorbic acid and tocopherol were shown to reduce oxidative damage. Moreover, the use of non-vitamin antioxidants (antiradicals) such as lipoic acid, glutathione, glycine betaine, and polyvinylpyrrolidone is beneficial. These natural compounds showed interesting results for viability in *Rubus* spp. shoot tips, which increased by 25% after LN storage [122].

The use of NPs in recent years has received attention since they are proving benefits to improve in vitro plant properties and regeneration rate, depending on their type, size, shape, and concentration [123,124]. It is presumed that the addition of NPs into the recovery medium could result in pores formation in the roots stimulating a greater uptake of water and, consequently, in significant plant growth. This phenomenon appeared in the case of manganese nanoparticles (MnNPs), which produced a differential change in the morphology and physiology of deadly nightshade (*Atropa belladonna* L.) with lower doses at 50 mg·L^−1^ [125]. Silver nanoparticles (AgNPs) were applied to rice (*Oryza sativa* L., cv. Swarna) and described as phytostimulants in some active compounds (i.e., chlorophyll and carotenoids) without negative impacts on the plant [126]. On the other hand, the NP excess could produce phytotoxicity, oxidative stress damage, and genotoxic effects [125,127]. For example, growth inhibition, especially in the root zone, was evident in wheat (*Triticum aestivum* L.) using copper nanoparticles (CuNPs) [128]. Overall, nanoparticles have become an attractive alternative to improve agrobiotechnological production; even so, there are still limitations that must be studied, analyzed, and controlled to successfully apply NPs during the pre- and *post*-LN storage steps.

## 10. Cryopreservation Applications of Agronomic Interest

Most studies related to cryopreservation are published with a protocol applicable for individual species and varieties or cultivars, as in the case of beetroot (*Beta vulgaris* L.) [129], ginger (*Zingiber officinale* Rosc.) [130], oca (*Oxalis tuberosa* Mol.) [131], or ulluco (*Ullucus tuberosus* Cal) [132], without maintaining a standard protocol for a large group, such as grains, fruits, and vegetables. However, a study published in 2017 was essential to adapt a basic protocol for the cryopreservation of a more diverse group of crops in the agronomic sector [133]. In this study, the shoot tips of four genera (*Malus*, *Solanum*, *Lonicera*, and *Berberis*) were tested with variations of cold acclimation, preculture media, and PVS2 exposure times. The use of the simple vitrification method based on a 0.3 M sucrose pretreatment and 30- (for potato) or 80-minute exposure to PVS2 (for apple and berry) showed a mean viability greater than 50%. This could greatly reduce some other unnecessary materials for use in cryopreservation banks.

Another successful protocol that has been used is encapsulation-dehydration with the use of LS composed of 0.8 M sucrose and 0.1 M glycerol (10–20 min exposure) and drying over a silica gel for 14 to 21 h. This protocol was applied to seeds of lettuce, Chinese kale, and ‘pak choi’ (a type of Chinese cabbage), which had a survival rate of 100%, being a great advance for the use of standard protocols useful in cryogenic banks. Two other vegetables, ‘Chinese cabbage’ and ‘White cabbage’ in bloom, were also studied and had a survival rate of 60−90.9% and 25−91.6%, respectively, and were able to grow in the ex vitro field conditions [134]. The same cryopreservation method was successfully used for in vitro-derived shoot apices of mint (*Mentha* × *piperita* L.) [103]. The use of PVS2 and PVS3 in cryopreservation of mint germplasm also showed suitable results of viability. However, some accessions, such as *M. requienii* and *M. villosanervata*, did not have an as good response with PVS2, highlighting the use of PVS3 in this plant genera for complete recovery of specimens [135]. 

The chili pepper seeds (Huareo n° 13) were cryopreserved with the vitrification method, by immersing them in an LS (composed of 0.4 M sucrose and 2.0 M glycerol) and in PVS2, applied for different durations (0, 10, and 20 min; 0, 30, and 60 min, respectively), showing a survival rate of 100%. These in vitro seedlings were successfully transplanted to field conditions [136].

Plants grown from seeds of corn (*Zea mays* L.) did not show a significant difference compared to those obtained from seeds previously stored in cryopreserved germplasm banks. The germination after LN storage was almost total and complete. Thus, a cryobank of maize landraces from two regions of Costa Rica was established [137,138].

One of the largest agronomic economies is managed by the winemaking sector. The maintenance and in vitro cultivation of grapevine (*Vitis* spp.) is highly expensive due to the specific growth requirements. Therefore, a cryopreservation protocol of this fruit crop was developed. Apical shoot tips (1 mm long) were pre-treated for three days on MS medium containing 0.3 M sucrose, salicylic acid (SA), glutathione, ascorbic acid, and plant preservative mixture. Half-strength PVS2 was applied for 30 min at 22 °C, prior to full-strength PVS2 treatment at 0 °C for another 30 min. This procedure achieved high levels of regrowth (55%) in *V. vinifera* ‘Chardonnay’ and ‘Riesling’, as well as *V. hybrid* ‘Oppenheim’. Based on the histological observations, it can be deduced that this positive response was related to the use of apical tips with multiple lateral meristems that survived LN immersion [139].

Onions are another important food source in global agronomic production, for which cryopreservation protocols have been developed. One of them was based on the droplet-vitrification method, applied for shallots (*Allium cepa* ‘Aggregatum’). Maintenance of in vitro cultures was performed in MS medium supplemented with PGRs, such as BA and 1-naphthaleneacetic acid (NAA). For cryopreservation, shoot tips were precultured with 0.3–0.5 M sucrose for 1 day followed by a loading phase in LS (composed of 0.6 M sucrose and 2.0 M glycerol) and dehydration in PVS3 (20 min and 3 h). This approach produced a 94% survival rate and 58% recovery rate. The cryopreservation-derived plant material showed no significant genetic difference from the non-cryopreserved control, nor changes in the content of components such as sugars and flavonoids [47,140]. In the ‘Kverve’ and ‘Lunteviga’ cultivars, the same steps of the droplet-vitrification method were applied. As a result, regrowth of 45% and 70% explants was observed. Both studies concluded that with onion, better results are obtained with explants treated with PVS3 than with PVS2, as the former one is less toxic to cells and provided a broader safe temperature range (−196 °C to −88 °C), compared to that (−196 °C to −116 °C) of PVS2 [47,141].

As for garlic (*Allium sativum* L.), the efficiency of cryopreservation depends on the origin of the donor explant. According to Keller [142], in vitro-grown material is the least responsive one concerning the regrowth rates. However, *post*-harvest storage duration of bulbs dramatically influences survival and recovery levels of cryopreserved shoot tips, which were nil for samples cryopreserved immediately after harvest and highest after 3 and 6 months of storage [38]. The field performance of LN-recovered garlic plantlets under ex vitro conditions was compared with garlic derived from the field, evaluating parameters such as net photosynthetic rate, bulb characteristics, and efficiency of eradication of yellow onion dwarf virus (OYDV) induced by cryotherapy. The superiority of the morphological traits of cryopreservation-derived garlic was evident, with greater size and weight of bulbs, in addition to a virus elimination rate of 75%. These results are relevant for the future application of cryopreservation to obtain products of better quality for the agronomic sector [141]. According to Kim et al. [143], the use of PVS3 is the most appropriate for the cryopreservation of garlic. Vitrification of seven garlic cultivars with PVS3 for 150–180 min ensured 92% recovery after rewarming [38]. The droplet-vitrification method was also successfully applied with several *Allium* germplasm collections, indicating that it can be used on a large scale to carry out international exchanges and with great agronomic potential [45,144]. On the other hand, the use of physical/air dehydration in this species has been shown to be harmful, as it radically decreases cryopreservation success. In parallel, it was shown that the addition of PGRs into the recovery medium, such as zeatin (ZEA) and gibberellic acid (GA), would significantly increase the final fresh weight of the LN-recovered plantlets [38].

The potato is thoroughly studied as one of the main agronomic crops in the world [145]. There are over 4000 varieties and cultivars of this species developed over time by breeding programs. Moreover, depending on the area of cultivation, the species has developed genetic diversity through adaptation to different (a)biotic conditions [39]. To preserve this genetic variability, it is necessary to make use of vitrification-based cryopreservation methods.

The effect of the subculture conditions of the mother plant of cryopreserved potato (‘Dejima’, cultivated; ‘STN13′, wild) was studied by Yoon et al. [45] using the droplet-vitrification method. It was observed that the studied conditions such as light intensity, aeration, and planting density significantly affected the survival of the cryopreserved and non-cryopreserved shoot tips, in both genotypes. Combinations of high light intensity, ventilation of culture vessels, and low planting density enhanced the viability of cryopreserved shoot tips (over 70%), with aeration being the most critical factor. The duration of the subculture and the location of the explant on the microshoot had a significant effect on the cryopreservation efficiency. The ideal duration of the subculture was 7 and 5 weeks and the optimal shoot tip size was 1.5 to 2.0 mm and 1.0 to 1.5 mm for ‘Dejima’ and ‘STN13′, respectively. The highest and lowest survival rates were found in explants sampled from the middle and apex parts of mother plants, respectively, in both cultivars studied. The survival of cryopreserved explants was influenced by the concentration of sucrose in the PC medium and the duration of the PC. The highest survival of cryopreserved shoot tips (91.9% for ‘Dejima’ and 86.4% for ‘STN13′) was observed when PC with 0.3 M sucrose for 8 h was followed by 0.7 M sucrose for 18 h and with PVS2 treatment for 20 min. These results indicate that the parameters of the subculture of the mother plant and the preculture of the explants must be carefully optimized, especially in the case of wild varieties.

The ‘Criolla’ potato (*Solanum tuberosum* Group Phreja) was studied through the encapsulation-dehydration method, using calcium alginate as an encapsulating matrix and silica gel for desiccation [146]. In this study, the effect of various sugars added into the PC medium was evaluated. The highest regrowth rates of 88% and 91% were reported when maltose and trehalose were applied, respectively, indicating those compounds as the most effective cryoprotectants. The viability of potato shoot tips in the presence of polyols was very low and no recovery was obtained after cooling of explants that were precultured in monosaccharides. The authors also demonstrated a significant effect of PC medium composition on the total soluble carbohydrate content in the cells. Another study was performed with the ‘Climax’, ‘Avon’, and ‘Ceza’, in which a direct relationship was obtained between the concentration of sucrose and the time in the PC medium to achieve higher viability [147].

The first purple-fleshed potato cryopreservation experiment by Li et al. [104] was using the encapsulation-vitrification and the droplet-vitrification methods. The duration of PVS2 dehydration influenced the survival rate, being the highest after 5–7 h and 6 h treatment for ‘E03-2677′ and for ‘Blue Congo’ cultivars, respectively, in the encapsulation-vitrification technique, and 30–50 min and 40 min, respectively, in droplet-vitrification. Vegetative growth in regenerated shoots after three weeks of *post*-rewarming culture was significantly lower than that of the control, but markedly increased in the following six months of culture.

Studies on *Solanum juzecpzukii* ‘Piñaza’, a species tolerant to low temperatures, and *Solanum tuberosum* spp. andígena ‘Ccompis’ and *S. tuberosum* ‘Désirée’, which are drought-tolerant, gave a positive response to the use of the droplet-vitrification method. The following parameters were selected as optimal: explants at three weeks of age were exposed for 15 min to LS and 50 min to PVS2. This method showed a significantly higher recovery than the original IPC cryobank method (20 min in PVS2) [44]. In parallel, *Solanum tuberosum* was studied using 10% DMSO as a cryoprotectant, obtaining a mean survival rate of 72%, in addition to an average recovery rate of 48% [148].

Potato viruses can cause significant problems in field cultivation. Among them, potato virus S is one of the most difficult pathogens to eliminate in agricultural practice. Cryo-treatment can be an effective tool in eradicating viruses from vegetatively propagated species. Kushnarenko et al. [149] described the effect of combined ribavirin treatment and cryotherapy for efficient eradication of five potato viruses (potato leaf virus, potato M, S, X, and Y viruses). Ruiz-Sáenz et al. [5] studied the effect of SA as a pre-treatment in the cryotherapy of seedling clones of *S. tuberosum* ‘Désirée’, which are drought-tolerant, gave a positive response to the use of the droplet-vitrification method. The following parameters were selected as optimal: explants at three weeks of age were exposed for 15 min to LS and 50 min to PVS2. This method showed a significantly higher recovery than the original IPC cryobank method (20 min in PVS2) [44]. In parallel, *Solanum tuberosum*, and the method will be adapted to other potato cultivars prone to contagion. Moreover, Ayala-Hernandez et al. [150] demonstrated that SA induces stress tolerance to which explants are subjected during cryopreservation. Pretreatment with 10^−6^ and 10^−5^ M SA provided significantly greater survival of explants to cryogenics (2.17–3.21 times with respect to the control).

Some recently developed cryoprotocols applicable to various plant species are shown in Table 4, indicating the droplet-vitrification method as the most common one with agronomic plants, and the *Solanum* genus as the most well-studied in terms of long-term storage.

**Table 4:** Recently developed cryopreservation protocols of selected crops of agronomic interest.

Biological Material	Explant	Preculture	Pretreatment	Storage and Rewarming	Recovery	S(R) (%)	Remarks	Ref.
*Vitrification*								
*Allium cepa* var. *aggregatum* (Shallot ‘10603’)	Shoot tips (2–3 mm) with 4–5 leaf primordia	MS medium + 30 g/L sucrose, 0.5 mg/L BA, 0.1 mg/L NAA and 8 g/L agar (pH, 5.8)	PVS3 at 24 °C for 3 h	LN storage for 1 h	Preculture medium for 8 weeks	>95	Rooting, vegetative growth, bulb production, genetic stability, and biochemical compounds were maintained after LN storage.	[151]
*Allium sativum* ‘Gailiangsuan’ cv. G064	Shoot apices (2 mm)	MS medium with 6.5 g/L agar and 0.5 M sucrose for 4 d at 23 °C and 12 h photoperiod	LS: 18.4% glycerol + 20.5% sucrose in MS medium without agar for 20 min at 24 °C. PVS2 for 30 min at 0 °C	LN storage for 1 h. Rapid rewarming by directly plunging the samples into RS for 10 min at RT	B5 + 0.1 mg/L NAA + 2.0 mg/L 6-BA, with 30 g/L sucrose, 6.5 g/L agar in the dark for 4 d	82.6 (75.9)	The LN-recovered plants were stable at the genetic and structural levels.	[152]
*Solanum tuberosum* ‘Superior’	Shoot tips (2–3 mm)	Liquid MS medium with 0.3 M sucrose on a rotary shaker at 60 rpm for 24 h	LS: AFP III, (0–2000 ng/mL) + liquid MS medium, 0.6 M sucrose, 2.0 M glycerol at 25 °C for 1.5 h. Or PVS2 with AFP III for 30 min at 25 °C	LN storage for 30 min in and at −20 °C for 1 h. Rewarmed in a water bath (38 °C) for 2 min, and washed for 20 min in NH_4_^+^-free MS medium with 1.2 M sucrose at 25 °C	MS medium with 88 mM sucrose, agar 8 g/L	26–39 (12–30)	This finding suggests that AFP increased cryopreservation efficiency by transcriptional regulation of these genes, which might protect plant cell membranes from cold stress during cryopreservation.	[153]
*Solanum tuberosum* (28 genotypes)	Apical shoots (2–3 mm)	16–20 h in liquid MS medium with 0.09 M sucrose	liquid MS medium with 0.09 M sucrose and 10% DMSO for 2 h at RT	LN storage for 1 h. Rewarming in a water bath at 40 °C	MS medium with 0.09 M sucrose, 0.5 mg/L ZR, 0.2 mg/L GA, 0.5 mg/L IAA at 25/20 °C-d/n temperature 16 h photoperiod at low light intensity	(54)	All 28 genotypes had higher regrowth after cryopreservation using PVS3 instead of DMSO.	[154]
*S. tuberosum* ‘Zihuabai’	Nodal segments (1 cm)	MS with 0.45 M sucrose in the dark at 5 °C for 1 d	60–80% PVS2 for 30 min and 100% PVS2 for 40 min at 0 °C	LN storage for 1 h. Rewarming in a water bath at 38 °C for 2 min and then in RS at 25 °C for 20 min	MS with 0.5 mg/L IAA, 0.5 mg/L ZR and 0.2 mg/L GA and kept in the dark at 22 ± 2 °C for 3 d	~80 (45.5)	No genetic alterations were detected in the recovered shoots by ISSR and AFLP.	[39]
*Vitis vinifera* L. ‘Flame Seedless’	Axillary buds	MS medium	PVS2 at 25 °C with agitation for 3 h	LN storage for 1 h, 1 week, and 1 month. Rewarming in a water bath at 38 °C for 3 min	NR	NR	Cryopreservation affect genetic stability in grapevine, regardless of storage duration.	[155]
Droplet-Vitrification								
*Allium cepa* ‘Kverve’ ‘Lunteviga’	Shoot tips (2–3 mm), 4 weeks old	MS medium with 0.3 M and 0.5 M sucrose, 1 d each	LS: 2.0 M glycerol + 0.6 M sucrose (20 min) and PVS3 at 24 °C (3 h)	LN storage for 1 h. Rewarming in RS at 25 ^°^C for 20 min	MS medium with 0.3 M sucrose for 2 d in the dark and then transferred to light	(45–70)	PVS2 was more effective than PVS2 in securing the explants. Cryopreservation of shoot tips was more effective than of meristemoids.	[140]
*Allium cepa* var. *aggregatum*	Shoot tips (2–3 mm)	MS supplemented with 30 g/L sucrose, 0.5 mg/L BA, 0.1 mg/L NAA and 8 g/L agar	PVS3 at 24 °C for 3 h	LN storage for 1 h	Preculture medium for 8 weeks	NR	No differences in rooting, vegetative growth, bulb production, and contents of soluble sugars and flavonols between the cryo- and in vitro-derived plants. No polymorphisms found in the cryo-derived plants by ISSR and AFLP markers.	[151]
*Helianthus tuberosus* ‘M6′ ‘Relikt’ ‘Shudi’ ‘Stampede’	Shoot tips (2–3 mm)	Liquid MS medium with 0.4 M sucrose (3 d)	LS: 2.0 M glycerol + 0.4 M sucrose (30 min) and PVS2 at 0 °C (15 min)	LN storage for 1 d. Rewarming in RS at 25 °C for 20 min	MS medium with 0.29 µM GA, 15% sucrose, and 8% agar cultured in the dark for 3–5 d, and then under 14 h photoperiod	93 (83)	Minimal cellular damage observed within the meristem cells of the shoot tips. No polymorphism detected by SSRs.	[62]
*Oxalis tuberosa* and *Ullucus tuberosus*	Shoot tips (2 mm)	MS semisolid medium with 2% sucrose, 2 mg/L calcium pantothenate, 10 mg/L putrescine, 0.25 mg/L GA for oca; and with 2% sucrose and 2 mg/L calcium pantothenate for ulluco	LS: 2 M glycerol and 0.4 M sucrose in MS medium at RT for 20 min. PVS2 for 60 min, at 0 °C	LN storage for 30 min. Rewarming in RS for 20 min at RT	MS with, 0.3 M sucrose, 0.04 mg/L KIN, 0.1 mg/L GA, and 0.28% phytagel, incubated in the dark for 2 d. Then, 0.1 M sucrose/dark/2 d. Finally, MS medium with 0.07 M sucrose+ 2 m /L calcium pantothenate at 20 °C	15 and 35, respectively	The protocols efficiency requires further improvement.	[131]
*S. tuberosum* ‘Agrie Dzeltenie’ ‘Anti’ ‘Bintje’ ‘Désirée‘ ‘Maret’	Shoot tips (1–3 mm)	MS solution with 3% sucrose	10% DMSO in liquid MS	LN storage for 1 h. Rewarming at RT in liquid MS	MS medium with 0.5 mg/L zeatin riboside, 0.2 mg/L GA, 0.5 mg/L IAA, 30 g/L sucrose	17.1–52.6	The optimization of light spectra during the recovery phase is a promising tool for increasing the recovery of potato shoot tips after cryopreservation.	[156]
*S. tuberosum* ‘Désirée’ *S. commersonii* Dun.	Shoot-tips (1 × 0.5 mm), 3 weeks old	MS medium with 0.21 M sucrose at 6 °C, 16/8-hour d/n and a light intensity of 50 μmol m^−2^ s^−1^ for 2 weeks	LS: 2 M glycerol and 0.4 M sucrose in MS medium (20 min). PVS2 for 50 min at 0 °C	LN storage for 30 min. Rewarming at RT in RS (20 min)	MS medium with 0.3 M sucrose (1 d). and then 0.09 M sucrose in the dark for the first 7 d	>80	First study in which cryopreservation experiments are combined with the observation of the responses to abiotic stress exposure.	[157]
*S. tuberosum* ‘Zihuabai’	Nodal segments (1 cm)	MS with 0.3 M sucrose in the dark at 5 °C for 3 d	LS: 2 M glycerol and 0.4 M sucrose in MS. PVS2 at 0 °C for 40 min	LN storage for 1 h. Rewarming in a water bath at 38 °C for 2 min and then in 1.2 M sucrose at 25 °C for 20 min	MS supplemented with 0.5 mg/L IAA, 0.5 mg/L ZR and 0.2 mg/L GA and kept in the dark at 22 ± 2 °C for 3 d	~80 (72.5)	No genetic alterations were detected in the recovered shoots by ISSR and AFLP.	[39]
*S. ajanhuiri* ‘Wila Yari’ CIP702650 *S. commersonii* CGN18024, *S. juzepcukii* ‘Piñaza’ CIP702445 S. *tuberosum* ‘Désirée’ CIP800048	Shoot tips (1 × 0.5 mm)	MS medium with 0.3 M/0.09 M sucrose at 6 °C for 14 d	LS: 2 M glycerol and 0.4 M sucrose in MS for 20 min in the dark, at RT. PVS2 for 50 min at 0 °C	LN storage for 30 min. Rewarming in RS at RT (20 min)	MS medium with 0.3 M sucrose solidified with 0.25% gerlite for 1 d and transfer to MS with 0.09 M sucrose	90–100	The increased accumulation of sucrose and raffinose family of oligosaccharides play a fundamental role in the response to stress in potato and may help to acquire tolerance to cryopreservation.	[158]
*S. commersonii, * *S. tuberosum* spp. andigena, *S. tuberosum* spp. tuberosum, *S. × ajanhuiri,* *S. × juzepczukii*	Shoot tips (1.8–2.5 mm), 3 weeks old	MS salts with 0.04 mg/L KIN, 0.1 mg/L GA, 0.07 M or 0.03 M sucrose and 2.8 g/L Phytagel at 6 °C and RT for 1 h	LS: 2 M glycerol and 0.4 M sucrose for 15 min at RT PVS2 for 50 min on ice	LN storage for 1 h. Rewarming in RS at RT (15–20 min). in the dark	MS salts with 0.04 mg/L KIN 0.1 mg/L GA, 2.8 g/L phytagel + 0.3 M sucrose. Daily transfers from 0.3, to 0.2, to 0.1 M and finally maintained on 0.07 M sucrose at 22 °C, 16 h photoperiod	40–100	This method is recommended for the long-term conservation of diverse accessions of potato germplasm.	[99]
*Solanum tuberosum* ‘Agrie’ ‘Anti’ ‘Bintje’ ‘Désirée’ ‘Dzeltenie’ ‘Maret’	Shoot tips (1–3 mm)	MS medium with 2% sucrose and 6.4 g/L agar under various light spectra conditions at 22 °C	LS: MS-solution with 3% sucrose under the original light quality treatments overnight. Dehydration for 2 h in MS-solution with 10% DMSO	LN storage for 1 h. Rewarmed by dipping the foils with shoot tips into 30 mL of MS solution at RT	MS medium with 0.5 mg/L ZR, 0.2 mg/L GA, 0.5 mg/L IAA, 30 g/L sucrose and (1 mL /L) PPM	31–66 using blue light	Light spectral quality before cryopreservation can significantly affect the cryopreservation success of potato shoot tips.	[119]
*S. tuberosum* ‘Blue Congo’ ‘E03-2677′	Buds (1.0–1.4 mm)	MS with 0.3 M sucrose at 4 °C and 16 h photoperiod for 3 weeks	LS: MS with 0.1 M sucrose and 2 M glycerol for 30 min on ice. PVS2 for 30 to 40 min on ice	LN storage for 1 h. Rewarming in a water bath at 38 °C for 2 min	MS medium with 0.2 mg/L GA, 0.5 mg/L IAA, and ZR for 3 d in the dark. Then, MS with 0.05 mg/L GA in light	(70–80)	First report on cryopreservation of purple-fleshed potato by vitrification-based procedures. Larger explants (1.5–2.0 mm) were less effective.	[104]
*S. tuberosum* (28 genotypes)	Shoot apices (2–3 mm)	Liquid MS medium with 0.3 M sucrose for 16–20 h	LS: 0.4 M sucrose and 2 M glycerol in MS for 20 min and PVS3 for 2 h	LN storage for 1 h. Rewarming at 40 °C, unloading in RS (20 min) recovered 25/20 °C d/n or only 20 °C for 7 d	MS medium with 0.09 M sucrose, 0.5 mg/L ZR, 0.2 mg/L GA, 0.5 mg/L IAA at 25/20 °C d/n temperature in the dark (7 d)	(71)	All 28 genotypes had higher regrowth after cryopreservation using PVS3 instead of DMSO.	[154]
*Potato varieties (Solanum spp.)*	Shoot tips with 3–4 leaf primordia (length: 0.8–1.2 mm; width: 0.4–0.7 mm)	MS medium with 0.04 mg/L KIN, 0.1 mg/L GA, 25 g/L sucrose and 2.8 g/L Phytagel	LS: 2.0 M glycerol and 0.4 M sucrose in MS at RT for 20 min. PVS2 on ice for 50 min	LN storage for 24 h. Rewarming in liquid MS medium with 0.0–1.2 M sucrose for 20 min at RT	MS medium with 0.4 mg/1 KIN, 0.1 mg/L GA, 20 mL /L coconut water, 0.3 M sucrose, 2.8 g/L Phytagelfor 4 d under diffuse light at 18–22 °C with a 16 h photoperiod	74.6–90.7 (66.5–86.8)	The specific response to low (0.0 M) and high (1.2 M) concentrations of sucrose in the unloading solution was highly variable within species/subspecies and appears to be genotype-specific.	[67]
*Encapsulation-Vitrification*								
*Solanum tuberosum* ‘Zihuabai’	Nodal segments (1 cm)	MS with 0.3 M sucrose (1 d) and then suspended in MS with 2.5% (*w*/*v*) alginate and 0.4 M sucrose	LS: 2 M glycerol and 0.6 M sucrose in MS for 90 min and then dehydrated with PVS2 at 0 °C for 4 h	LN storage for 1 h. Rewarming in a water bath at 38 °C for 2 min and then in 1.2 M sucrose at 25 °C for 20 min	MS supplemented with 0.5 mg/L IAA, 0.5 mg/L ZR and 0.2 mg/L GA and kept in the dark at 22 ± 2 °C for 3 d	70	No genetic alterations were detected by ISSR and AFLP.	[39]
*Solanum tuberosum* ‘Blue Congo’ ‘E03-2677′	Buds of 1.5–2.0 mm	MS with 0.3 M sucrose at 4 °C and 16 h photoperiod for 3 weeks, then suspended in 1/2 MS with 3% alginate, 2 M glycerol, and 0.4 M sucrose	MS containing 2 M glycerol and 0.6 M sucrose at RT for 90 min and PVS2 on ice for 6–7 h	LN storage for 1 h. Rewarming in a water bath at 38 °C for 2 min	MS medium with 0.2 mg/L GA, 0.5 mg/L IAA, and ZR for 3 d in the dark. Then, MS with 0.05 mg/L GA in light	(40–80)	First report on cryopreservation of purple-fleshed potato by vitrification-based procedures. Smaller explants (1.0–1.4 mm) were less effective.	[104]
*S. tuberosum* ‘Zihuabai’	Nodal segments (1 cm)	MS with 0.45 M sucrose in the dark at 5 °C for 1 d	LS: 2 M glycerol and 0.6 M sucrose in MS for 90 min. PVS2 at 0 °C for 4 h	LN storage for 1 h. Rewarming in a water bath at 38 °C for 2 min and then in 1.2 M sucrose at 25 °C for 20 min	MS with 0.5 mg/L IAA, 0.5 mg/L ZR, 0.2 mg/L GA, kept in the dark at 22 °C for 3 d	~80 (70)	No genetic alterations detected in the recovered shoots by ISSR and AFLP markers.	[39]
*Encapsulation-Dehydration*								
*A. officinalis* ‘Morado de Huétor’	Rhizome buds	MS with 0.3 M sucrose at 25 °C for 24 or 48 h	Explants embedded in alginate (3%), sucrose (0.4 M) and glycerol (2 M). Desiccation over silica gel for 24 h	LN storage for 24 h. Rewarming in distilled water at RT	MS modified with Ferric Sodium EDDHA 85.7 mg/L, 0.5 mg/L NAA, 0.7 mg/L kinetin, 2 mg/L Ancymidol, and 6% sucrose	34.5–84	Confirmation of ploidy and molecular stability of LN-recovered plantlets.	[50]
*Mentha* × *piperita* ‘MEN 198′	Axillary buds (1–2 mm)	MS medium with 0.3 M sucrose for 1 d at RT and 16 h photoperiod at low light intensity. Explants suspended in MS with 3% alginate and 0.35 M sucrose (30 min)	Osmotic dehydration in MS liquid + 0.75 M sucrose at 120 rpm for 18–20 h. Then, drying in a flow chamber for 5 h to a level of 22% initial water content	LN storage for 1 d. Rewarming in a water bath at 40 °C for 2 min	NR	NR	RAPD and AFLP showed an almost complete genetic stability of the recovered plants.	[103]
*Solanum phureja*	Single node stem cuttings (1 cm)	0.1 M sucrose in TR medium, MS microelements, vitamins of MW medium for 7 d at RT, and 16 h photoperiod. Suspension in 3% alginate and 0.1 M sucrose	PC medium supplemented with (sugars and polyols) (0.65 M) for 2 d at RT. Dehydration for 4.5 h on silica gel to a level of 22% initial water content	LN storage for 1 h. Rewarming at RT	PC medium	88 (maltose) and 91 (trehalose)	Maltose and trehalose were the most effective cryoprotectants. Shoot tips precultured in sucrose, trehalose or glucose, indicated an increase in total soluble sugars, especially when sucrose was applied as a cryoprotectant.	[146]
*Cryo-plate*								
*Allium* spp. *A. sativum * *A. fistulosum* var. caespitosum *A. chinense*	Shoot tips (2.5 mm), 2 days old	½MS medium with 0.3 M sucrose for 2 d. Explants embedded on cryo-plates with 3% alginate and 0.4 M sucrose (15 min)	LS: 1.0 M sucrose and 2.0 M glycerol (30 min) at 25 °C. Air desiccation for 30 to 180 min	LN storage for 1 h. Rewarming in 1.0 M sucrose solution in 1/2 MS medium at 25 °C for 30 min	½MS medium with 3.0% sucrose and 0.8% agar	94	The glass transition temperature of shoot tips after air desiccation was −39.4 °C.	[159]
*Solanum tuberosum* ‘Sayaka’	Shoot tips (0.5–1.5 mm)	MS medium + 0.3 M sucrose and 0.3% gellan gum at 25 °C overnight. Explants embedded on cryo-plates with 2% alginate (15 min)	LS: 2.0 M glycerol and 0.8 M sucrose in MS at 25 °C for 30 min. V cryo-plate: PVS2 at 25 °C for 30 min D cryo-plate: air desiccation for 2 h	LN storage for 30 min. Rewarming in MS medium with 1 M sucrose for 15 min at RT	PC medium	D cryo-plate: 93.3; V cryo-plate: 96.7	Both protocols will facilitate efficient strategies for the preservation, storage, and maintenance of genetic stability of potato germplasm.	[160]
*Ullucus tuberosus* 11 lines	Shoot tips (1.0–1.5 mm)	MS with 0.3 M sucrose for 16 h at 25 °C	D cryo-plate: Alginate with 0.4 M sucrose. Cryo-plates + 2 M glycerol + 1 M sucrose for 90 min at 25 °C. Dehydration at 25 °C for 45 min	LN storage	-	73–97	D cryo-plate is a practical and simple procedure for cryostorage of in vitro grown ulluco shoot tips in an ex situ genebank.	[132]
*Desiccation*								
Potato: ‘Avon’ ‘Ceza’ ‘Climax’	Microtubers (~2 mm)	MS medium without vitamins with 100 g/L sucrose, and 10 g/L agar	Microtuber desiccation using sterile dry silica gel for 3–6 h (17–36% FW)	LN storage for 10 min. Rewarming in a water bath at 45 °C for 60 s and in a liquid MS medium at RT for 5–10 min	MS with vitamins, 30 g/L sucrose and 2 g/L activated charcoal at 25 °C with 16 h photoperiod	100	The desiccation technique is a simple approach for cryostorage of microtubers.	[147]
S(R) = Survival (recovery). NR = not reported. RT = room temperture. RS = rewarming solution. d/n = day/night.								

AFLP, amplified fragment length polymorphism; AFP, antifreeze protein; DMSO, dimethyl sulfoxide; ISSR, intersequence simple repeats; LN, liquid nitrogen; LS, loading solution; MS, Murshige and Skoog [90] medium; MW, Morel and Wetmore [161] medium; PC, preculture; PVS, plant vitrification solution; RAPD, randomly amplified DNA; RS, Sakai’s unloading solution [28]; TL, Tendille and Lecerf [162] medium.

## 11. Oxidative Stress Markers in LN-Derived Plant Material

Managing oxidative stress is vital for the successful application of cryopreservation to plant tissues. Freezing injury induces the production of free radicals, mainly ROS, which attack the lipid fraction of membranes [112]. Even pre-LN storage steps, such as hardening and dehydration, can induce excess ROS such as hydroxyl radicle, superoxide, and hydrogen peroxide, which can accumulate and cause severe damage to plant tissues [163]. Lipid peroxidation is well documented as occurring under cryo-stress. Therefore, the levels of enzymatic and non-enzymatic oxidative markers should be measured when developing a new cryoprotocol. 

In several plant species, including corn (*Zea mays* L.)—one of the most important grains in the world—it was reported that there is a strong relationship between the viability of LN-stored cells and the activity of superoxide dismutase (SOD), glutathione reductase (GR), peroxidase (POD), catalase (CAT), and ascorbic acid peroxidase (APX) [163,164,165]. As for kiwi (*Actinidia chinensis* Planch.), the presence of compounds known as protein carbonyls (PCO) was significantly affected by the cryopreservation procedure, leading researchers to conclude that PCO could serve as a marker for protein oxidative damage [101].

To better understand the possible reasons for a reduction in the survival level at the subsequent steps of cryopreservation, Subbarayan et al. [166] measured the concentration of amino acids, ammonium, γ-aminobutyric acid (GABA), soluble sugars, malondialdehyde (MDA), and oxygen in garlic shoot tips undergoing cryopreservation. They found an altered modulation in alanine and glutamate metabolism at the dehydration step that may indicate hypoxic stress in the tissue. Furthermore, the accumulation of MDA was confirmed to play an important role in cryo-stress, indicating that the generation of ROS is highest after the hypoxic dehydration step. Further studies are needed that examine hypoxia during dehydration at the molecular level.

## 12. Stability and Functional Genomics of LN-Derived Plant Material

While cryopreserving genetic resources, emphasis has always been given to the genetic integrity of the stored biological material. This is particularly important in the commercial production of certified elite plant material. It is assumed that once cryopreserved, biological samples remain safe and stable. Therefore, novel, mutant, or transformed cell lines can be stored cryogenically to prevent their loss. Generally, there is consensus that genetic drift and mutation occurrence are minimized under cryogenics compared to when samples are maintained in an actively growing state for extended intervals [121]. For example, the study by Li et al. [104] showed no polymorphism in the LN-recovered plantlets of purple-fleshed potato after applying inter simple sequence repeat (ISSR) and randomly amplified polymorphic DNA (RAPD) markers. Similarly, no mutations were detected by sequence simple repeat (SSR) molecular markers in LN-derived Jerusalem artichoke (*Helianthus tuberosus* L.). As for the commercial sugarcane (*Saccharum* spp.) germplasm cryopreserved with two PVS-2-based procedures, 100% genetic homogeneity was detected by ISSRs from Halaii and H 83-6179 cultivars, whereas 98.5% genetic stability was detected from NG 57-024 cultivar (Kaya and Souza 2017). Similarly, no ploidy changes were detected by flow cytometry (FCM) in asparagus cryopreserved with the encapsulation-dehydration technique [50]. Nonetheless, the potential effects of cryostorage of explants and seeds on subsequent plant growth in ex vitro conditions must be established before routine implementation in cryobanks, especially as genomic or phenotypic variation may result from tissue culture procedures commonly used in cryopreservation techniques. The dehydration phase (both physical and chemical in PVS) is the most critical for the stability of the plant material, as observed with spring wheat (*Triticum aestivum* L.) and tomato (*Solanum lycopersicum* L.) [167]. The duration of LN storage may also affect the parameters of plant material. For example, different ISSR fragment patterns were recorded in vitrification-derived buds of grapevine as compared with the untreated control after storage for one hour, one week, and one month [155]. As for wasabi (*Wasabia japonica* Matsumu), only a minor change in DNA sequence was found after two years of LN storage (when using the same technique) compared to the in vitro-grow control. However, when comparing the control and cryo-derived plant material after 10 years of storage, more mutations were found in the latter one [168]. Therefore, the vitrification technique seems to be the least ‘safe’ for long-term germplasm storage. This is probably due to the lack of additional physical protection present in the encapsulation-based techniques or slower cooling and rewarming rates than in the droplet-vitrification or cryo-plate techniques.

Recent studies revealed that DNA methylation and histone acetylation patterns in plants may be affected by cryoprotectants and/or liquid nitrogen exposure [112]. This epigenetic variation may cause genomic changes when methylation-sensitive marker systems are employed. Maki et al. [168] reported methylation changes in the DNA of wasabi after long-term storage of shoot tips at −150 °C. Likewise, Ibáñeza et al. [103] found that mint apices sampled immediately after each step of the encapsulation-dehydration protocol showed increased epigenetic differences as the protocol advanced, compared to in vitro-grown control, particularly related to de novo methylation events. However, after one-day in vitro recovery, methylation status was similar to the control objects. Those authors developed a simple and fast method for the analysis of methylation-sensitive amplified polymorphism (MSAP) markers, based on R programming, to improve the quality of methylation data interpretation and graphical representation [103].

The structural and phytochemical profile of plant material can also be more or less reversibly altered by the cryopreservation process [112]. In LN-derived tomato plants, the levels of cell wall-linked, free, and total phenolics decreased significantly in roots, stems, and leaves after 7–21 days of cryo-storage. Conversely, the concentration of the mentioned above compounds increased when seeds were immersed in LN for 28 days compared to non-treated control [169]. As for LN-derived sorghum (*Sorghum bicolor* (L.) Moench.) seeds, at 0–14 days after rewarming, the content of chlorophyll *a* was significantly higher than in the control samples, which may be attributed to the activity of enzymes related to chlorophyll biosynthesis, such as δ-aminolevulinic acid dehydratase and protochlorophyllide reductase. On the other hand, seedlings derived from cryopreserved seeds showed higher SOD and POD activities. Contrastingly, LN-derived and control adult plants were comparable in terms of chlorophylls, proteins, enzyme activities, plant architecture, and yield components [170].

Plant cryobiology also seeks to understand the molecular processes that allow plants to survive low temperatures and to explain why some genotypes are less tolerant of cryopreservation stresses than others. Recent discoveries in functional genomics in *Musa* and *Arabidopsis* have revealed the involvement of genes and proteins in the glycolytic and other metabolic pathways engaged in dehydration tolerance, osmoprotection, and membrane transport leading to cold acclimation and freezing tolerance acquisition [121]. It was found that prolonged stress-related to dehydration-rehydration cycles and low temperature invoke secondary messengers such as calcium, ROS, and inositol phosphates. The cascades initiating from these signal molecules result in the expression of transcription factors (TF) that affect the expression of hundreds of stress response genes, such as chaperones, LEA (Late Embryogenesis Abundant), osmotin, antifreeze, mRNA binding, osmolyte biosynthesis, water channel proteins, sugar and proline transporters, proteases, and detoxification enzymes [121,171]. Moreover, the complex mechanism of stress response in plants is not only controlled by genes but also by other factors, such as hormones, circadian clocks, and light [171]. Progress in genomic research may unravel fundamental physiological responses of cells to extreme conditions (including CPAs treatment, low temperatures, and in vitro recovery), the knowledge of which will guide cryobiologists in their quest to design improved long-term preservation strategies.

## 13. Conclusions

Cryopreservation methods are used by world germplasm banks to preserve the biodiversity of vegetatively propagated plant species of agronomic importance (and/or those with recalcitrant seeds) as a reference/base collection for the available genetic diversity of a population or as a source for new alleles in the future breeding programs. The cryoprotocols are being constantly improved to increase cell viability, eradicate viral diseases, and maintain the genetic stability of the germplasm. Recently, the field of plant cryobiology has advanced significantly through the use of molecular techniques. The challenge is to achieve the maximum versatility possible, which is why more studies must be carried out to further decrease the cytotoxicity of cryoprotectants, in addition to creating new standard protocols applicable to a variety of germplasms of worldwide interest. Studies on the inclusion of antioxidants should be carried out to obtain better results of viability. The application of NPs and natural animal and/or plant extracts can help achieve this goal. Moreover, the characterization of specific genes and proteins involved in dehydration tolerance, osmoprotection, and membrane transport in tissues will lead to significant advances in plant cryobiology research.

## Figures and Tables

**Table 1:** Synthesis of the essential steps involved in plant cryopreservation.

Cryopreservation Method	
Classic Methods (Slow Cooling)	Modern Methods (Rapid Cooling)
Establishment of in vitro culture or disinfection of tissues and organs taken directly from the donor plant	
Preculture of explants on a medium with an increased osmotic pressure and optional cold-hardening of explants	
Treatment with an appropriate cryoprotectant, usually DMSO (concentration and treatment duration vary depending on the plant material)	Dehydration with a mixture of diluted then concentrated cryoprotectants (after an optional encapsulation of explants in alginate) or dehydration in a series of sucrose solutions (with increasing concentration), followed by air drying
Gradual and slow cooling at a certain cooling rate (0.2–2 °C·min^−1^), which can be achieved by commercially available cryostats. This cooling is periodic up to −20 °C, −40 °C, −70 °C, −100 °C and, finally, −196 °C and at defined intervals	Fast cooling by direct immersion of the explants in LN
Storage of specimens in vials or straws in cryo-boxes in LN or, less often, its vapor phase	
Rewarming of samples (rapid in a water bath or at room temperature)	
Elimination of the cryoprotectant by washing with a solution of high sucrose concentration (usually 1.2 M)	
Determination of viability (histochemically or by growth observation)	
In vitro recovery of plants on a PGRs-supplemented media (usually at reduced light conditions during the first two weeks of culture)	
Acclimatization and transfer to ex vitro conditions	

**Table 2:** Composition of most common loading solutions (LS).

Solution	Component (% *w*/*v*)			Reference
	DMSO	Glycerol	Sucrose	
**LS1**	-	18.4	13.7	[72]
**LS2**	5.0	13.8	13.7	[73]
**LS3**	10.0	4.6	10.3	
**LS4**	10.0	-	24.0	

**Table 3:** Composition of plant vitrification solutions (PVS).

Solution	Component (% *w*/*v*)						Reference
	Sorbitol	EG	DMSO	PEG	Glycerol	Sucrose	
**PVS1**	9.1	15.0	7.0	15.0	22.0	-	[74]
**PVS2**	-	15.0	15.0	-	30.0	13.7	[75]
**PVS3**	-	-	-	-	50.0	50.0	[72]
**PVS4**	-	20.0	-	-	35.0	20.5	[37]
**L-solution**	-	30.0	7.0	-	22.0	15.0	[78]
**T-solution**	-	35.0	7.8	10.0	-	-	[79]
**W-solution**	18.7	-	44.5	-	-	-	[80]

## Data Availability

Not applicable.

## Abbreviations

ABA	Abscisic acid
AFLP	Amplified fragment length polymorphism
AFP	Antifreeze protein
APX	Ascorbic acid peroxidase
BA	N^6^-benzyladenine
CAT	Catalase
CBD	Convention on Biological Diversity
CIAT	International Center for Tropical Agriculture
CPAs	Cryoprotective agents
CRI	Crop Research Institute in the Czech Republic
DMSO	Dimethyl sulfoxide
DSC	Differential scanning calorimetry
EG	Ethylene glycol
EMBRAPA	Brazilian Agricultural Research Corporation
FCM	Flow cytometry
GA	Gibberellic acid
GABA	γ-aminobutyric acid
GR	Glutathione reductase
HEPES	4-(2-hydroxyethyl)-1-piperazine ethanesulfonic acid
InHORT	Research Institute of Horticulture in Poland
IPC	International Potato Center
IAA	Indoleacetic acid
ISSR	Inter simple sequence repeat
KIN	Kinetin
LEA	Late embryogenesis abundant
LN	Liquid nitrogen
LS	Loading solution
MDA	Malondialdehyde
MS	Murashige and Skoog
MSAP	Methylation-sensitive amplified polymorphism
NAA	1-naphthaleneacetic acid
NARO	National Agriculture and Food Research Organization in Japan
NCSS	National Center for Seeds and Seedlings in Japan
NIAS	National Institute of Agrobiological Sciences in Japan
NPs	Nanoparticles
PC	Preculture
PCO	Protein carbonyls
PEG	Polyethylene glycol
PG	Propylene glycol
PGRs	Plant growth regulators
POD	Peroxidase
PVS	Plant vitrification solution
RAPD	Randomly amplified polymorphic DNA
ROS	Reactive oxygen species
RS	Sakai’s unloading solution
SA	Salicylic acid
SOD	Superoxidase dismutase
SSR	Sequence simple repeat
SWCNTs	Single-wall carbon nanotubes
TF	Transcription factor
ZR	Zeatin riboside
